# The effects of two types of neighborhood factors on trajectory of internalizing and externalizing symptoms from early childhood to adolescence

**DOI:** 10.1371/journal.pone.0305632

**Published:** 2024-06-25

**Authors:** Fei Pei

**Affiliations:** School of Social Work, Falk College, Syracuse University, Syracuse, New York, United States of America; Yale University, UNITED STATES

## Abstract

Although a robust body of previous empirical studies investigated the long-term trend of child behavior problems, limited research discussed the influences of various types of neighborhood factors on such trajectory (e.g., neighborhood structural characteristics and collective efficacy). Using a nationally representative longitudinal dataset the Fragile Families and Child Wellbeing Study (FFCWS), with six waves from 1998 to 2017, this study captures the longitudinal effects of two types of early childhood neighborhood factors on the co-development of internalizing and externalizing symptoms. Data was collected at the focal child’s age 3, age 5, age 9, age 15 (N = 2,385), and the parallel-process growth curve models were applied. Results suggest that the trajectories of both internalization and externalizing symptoms showed U-shape and bidirectional relationships among internalizing and externalizing problems. The long-term effects of neighborhood social cohesion and economic disadvantages were significantly associated with children’s internalizing and externalizing symptoms. The implication of this study was further discussed.

## Introduction

From a developmental psychopathology perspective, the trajectory of child behavior problems, including internalizing and externalizing symptoms, is affected by various predictors [1–3], and early childhood behaviors can affect later developmental outcomes. Therefore, it is important to capture behavioral trajectories across different developmental stages. Although previous studies have suggested internalizing and externalizing symptoms often develop together, findings have been inconsistent and dated, with some suggesting that they reciprocally reinforce each other [4,5] and others reporting a unidirectional relationship [6,7]. Thus, clarifying the long-term relationship between internalizing and externalizing symptoms from early childhood to middle adolescence is important for future intervention and prevention programs.

Additionally, social ecological theory suggests that individual behaviors are influenced by factors across domains, including school, family, and neighborhood [8], yet previous studies haven’t discussed the influences of neighborhood factors on the trajectories of internalizing and externalizing symptoms. In particular, the influences of neighborhood structural factors and collective efficacy (referring to objective characteristics of people’s living environment and social connections among residents) on the trajectories of internalizing and externalizing symptoms remain unclear. The current study captured the trajectories of internalizing and externalizing symptoms from early childhood to middle adolescence and examined how the two types of early childhood neighborhood factors affect such trajectories.

### Trajectories of child behavior problems

According to developmental psychopathology and lifespan development perspectives [2,9,10], an individual’s development is a long-term process including origins, courses, and outcomes that feature various challenges and risks in different developmental stages. From such perspectives, early childhood experiences have long-term influences and affect children’s developmental outcomes in later stages. Behavioral problems can develop in early childhood and affect children’s behavioral problems in later developmental stages [11]. Child behavior problems can be categorized as internalizing and externalizing symptoms based on their characteristics [12,13]. Internalizing symptoms include internal feelings such as depression, anxiety, and social withdrawal. Externalizing symptoms refer to behaviors toward the external environment, such as aggressive and disruptive behaviors. Thus, internalizing symptoms and externalizing symptoms are the two closely related behavior problems that might be related to each other [13,14].

A large volume of research investigated the trajectories of internalizing and externalizing symptoms in different developmental stages separately, and studies have yielded inconsistent results [15–17]. Regarding internalizing symptoms, Sterba et al.’s study [15] following children from 2 to 11 years old suggested that internalizing behaviors followed either a low stable trajectory or a U-shaped curve, with decreasing symptoms in early childhood and increasing symptoms in later childhood. However, a meta-analysis of 310 studies found that depressive symptoms were stable in childhood and slightly increased between ages 12 and 16 [18]. Another empirical study discovered a trend of increasing internalizing symptoms among children from ages 6 to 11 [16]. Meanwhile, findings of the trajectory of externalizing symptoms have been even more inconsistent, with studies suggesting a simple decreasing trend, increasing trend, or stable trend in childhood and adolescence [19–21] and others indicating a nonlinear trajectory in childhood and adolescence [22,23]. Therefore, a comprehensive understanding of the trajectory of child behavior problems from early childhood to adolescence can expand the existing literature by providing evidence to clarify such trajectories.

### Trajectories and co-development of internalizing and externalizing symptoms

The co-development of internalizing and externalizing symptoms during childhood and adolescence has been commonly discussed in previous literature [13,17,24,25], yet mixed findings were presented regarding the direction and pattern of the trajectories of such symptoms. Many empirical studies reported increasing internalizing and decreasing externalizing symptoms over time [17,24,26,27]. For example, Papachristou and Flouri’s study [24] of 17,318 children between ages 3 and 14 found that high levels of externalizing symptoms were associated with a rapid increase in internalizing symptoms. Such results were supported by another study of 2,844 Korean children [26], wherein similar trajectories were found among children aged 10 to 14. Others reported relatively stable or decreasing trajectories of internalizing or externalizing symptoms over time [28]. Wiggins and his colleagues [29] suggested decreased trends of both internalizing and externalizing symptoms between ages 3 and 9.

Meanwhile, quadratic models were used in previous studies, and a curvilinear trajectory was reported [30–32]. An empirical study of 316 children between the ages of 4 and 10 indicated that internalizing symptoms increased between ages 4 and 8 or 9 and slightly decreased afterward, whereas externalizing symptoms decreased in this time period [31]. Another empirical study using quadratic models suggested that internalizing symptoms remained relatively stable between ages 4 and 12 and showed a slight decline around age 12, whereas externalizing symptoms decreased from ages 4 to 8 but then increased again [30]. Similarly, Cohen et al [33] suggested that internalizing symptoms decreased from age 7 to 12 and then increased going into adolescence. However, the research on the co-development of internalizing and externalizing symptoms is dated and further research with a larger sample size is needed to provide more conclusive findings regarding such trajectory.

Moreover, according to the developmental psychopathology theory, the development of internalizing and externalizing symptoms is a dynamic and interactional process across domains [10,34,35]. Internalizing and externalizing symptoms in different time courses of psychopathology can affect each other. Empirical findings regarding the directional relationships among internalizing and externalizing symptoms are inconsistent. Most research suggested a bidirectional relationship between internalizing and externalizing symptoms, suggesting they reinforce each other across domains [36,37]. Psychiatric research on the comorbidity of internalizing and externalizing symptoms suggested that such bidirectional relationships were due to shared etiological factors including a common genetic liability and environmental influences [38,39]. Other studies noted a unidirectional relationship between internalizing and externalizing symptoms, wherein some found internalizing symptoms predicted later externalizing symptoms and others suggested the opposite [6,26,40]. Theoretical models such as the classical theory of masked depression indicate that mental health issues can lead to conduct problems, because children express themselves through their behaviors [41]. Therefore, the cross-domain relationships among internalizing and externalizing symptoms require more investigation.

### Neighborhood factors and trajectories of internalizing and externalizing symptoms

According to Bronfenbrenner’s social ecology theory [8], children’s behaviors are affected by factors in the multilevel environment in which they live, including individual characteristics and family, school, and community factors. Commonly investigated predictors include caregivers’ mental health, parent—children attachment, and adverse childhood experiences [13,17,42]. Besides these commonly discussed predictors, the influences of neighborhood factors have been increasingly investigated by child behavior studies [43–45]. Neighborhood factors can be further categorized as neighborhood structural factors and collective efficacy based on their characteristics. Neighborhood structural factors reflect the objective characteristics of people’s living environment such as economic disadvantages, demographic characteristics, and residential instability [46]. Based on social disorganization theory, economic disadvantage, residential instability, and demographic characteristics can directly affect individual behaviors by creating a disadvantaged environment and lack of affluent community resources [46]. Neighborhood collective efficacy refers to residents’ perceptions of their living environment and social connections such as social cohesion and social control [47–49]. An example of neighborhood social cohesion is whether residents can get along with their neighbors. Social control pertains to whether residents would intervene when violent events happen in a neighborhood. Sampson and his colleagues [49] suggested that negative neighborhood collective efficacy can affect residents’ lives by reducing social support and trust among residents and undermining the control of disruptive behaviors in such neighborhoods.

Some empirical studies investigated the influences of different types of neighborhood factors on child behavior problems [45,50]. Wickrama and Bryant [50] suggested that high levels of neighborhood poverty and demographic characteristics were positively correlated with the occurrence of adolescent depressive symptoms between ages 13 and 19, but Moilanen et al. [40] found that neighborhood disadvantage was only related to externalizing symptoms from ages 6 to 12. Conflicting findings also emerged in empirical studies of neighborhood collective efficacy (e.g., social cohesion and social control) and behavioral problems [51]. Future research on how these two types of neighborhood factors work together to influence behavioral problems from early childhood to adolescence is needed.

Additionally, few existing studies have comprehensively investigated the influences of neighborhood factors on the long-term trajectory of the codevelopment of internalizing and externalizing symptoms, but some previous studies investigated specific facets of neighborhood factors and characteristics. A study of low-income urban youth between ages 10 and 15 found that community violence predicted the initial levels and declines of both externalizing and internalizing symptoms and showed stronger influences on externalizing symptoms than internalizing symptoms [52]. Another study of neighborhood collective efficacy (residents’ perceptions of their communities) found that better neighborhood quality was only associated with fewer externalizing symptoms among children aged 7 to 12 [53]. However, Riina et al.’s [54] study suggested that neighborhoods with higher social cohesion decreased the levels of internalizing symptoms for maltreated children aged 11 years or older. Additionally, the majority of previous empirical studies on internalizing and externalizing symptoms applied the variable-centered approach, which focused on understanding the relationship among variables. Some recent studies focused on identifying sub-groups of individuals based on their shared characteristics across a set of variables, utilizing the person-centered approach [55,56]. As the current study focused on the influences of two neighborhood factors on internalizing and externalizing symptoms, which is a variable-to-variable relationship, the variable-centered approach was applied.

To our knowledge, no national level study has systematically investigated whether these two neighborhood factors affect the trajectories of internalizing and externalizing symptoms from early childhood to adolescence. Comprehensively understanding the influences of neighborhood factors on the long-term trajectory of the codevelopment of internalizing and externalizing symptoms expands the knowledge of children’s living environment and provides further insights for neighborhood-level interventions and preventions.

### Present study

This study examines the long-term effects of early childhood neighborhood factors on trajectories of internalizing and externalizing symptoms from early childhood to adolescence. According to previous empirical research and the developmental psychopathology perspective, I hypothesized that: (1) the trajectories of internalizing and externalizing symptoms were nonlinear and the intercepts, slopes, and quadratics of internalizing and externalizing symptoms affect each other across domains; and (2) better neighborhood social cohesion and social control decreased the initial levels and slopes of internalizing and externalizing symptoms and living in disadvantaged neighborhoods increased the initial levels and slopes of internalizing and externalizing symptoms.

## Method

### Data and sample

This secondary data analysis used data from the restricted version of the Fragile Family Child Wellbeing Study (FFCWS), a longitudinal project that collected information about the well-being, behaviors, and parenting environment of 4,898 children, especially children born in disadvantaged families. Participants were randomly selected from 20 large American cities with populations larger than 200,000. Documents of these children were randomly selected from the local hospitals. Six waves of data were collected: baseline and Years 1, 3, 5, 9, and 15 (Waves 1–6, respectively). Baseline interviews were conducted in randomly selected hospitals after selected mothers gave birth. Data from the Year 3, 5, 9, and 15 (Waves 3–6) Primary Caregiver Survey and Core Survey were used in this study because these waves measured their neighborhood environment. In Wave 3, 3,288 caregivers provided information about their children and living environment. Sample sizes in Waves 4, 5, and 6 were 2,989, 3,630, and 3,580, respectively, and some missing values existed in each wave. The current study used data from four waves (Waves 3–6), and after using full maximum likelihood estimation to deal with missing data, the final sample size was 2,385. For the variables used in the current study, focal children’s demographic information was collected at baseline and Year 3. Neighborhood factors used in this study were measured at children’s age 3, and children’s behavior problems were measured at ages 3, 5, 9, and 15.

### Measures

#### Behavior problems

Internalizing and externalizing symptoms were measured at the focal child’s ages 3, 5, 9, and 15 using the Child Behavioral Checklist [57]. An 8-item internalizing subscale (including anxiety, depression, and social withdrawal) and a 17-item externalizing subscale (including aggression and rule-breaking behaviors) were used to construct the perspectives and understanding of internalizing symptoms and externalizing symptoms, respectively. Caregivers rated their children’s behaviors using a 3-point scale (0 = *not true*; 1 = *somewhat or sometimes true*; 2 = *very true or often true*). Cronbach’s alphas ranged from.74 to.79 for the internalizing scale and from.85 to.89 for the externalizing scale. Sum scores were created for internalizing and externalizing symptoms separately in each wave, and higher sum scores indicated more severe symptoms.

#### Neighborhood structural factors

Neighborhood structural factors were measured at children’s age 3, broken down into three components: economic disadvantage, residential instability, and demographic characteristics [46,58]. Using principal component analysis, economic disadvantage was indexed by five items: the percentage of non-Hispanic Black population (*M* = 39.35, *SD* = 36.59), percentage of families below the federal poverty line (*M* = 18.00, *SD* = 13.90), percentage of civilian labor force (aged 16 or older) that is unemployed (*M* = 10.00, *SD* = 7.30), percentage of population with an education less than a bachelor’s degree (*M* = 83.00, *SD* = 14.80), and percentage of households on public assistance (*M* = 7.00, *SD* = 6.60) [40,49,58]. Residential instability was measured by the percentage of renter-occupied homes (*M* = 54.00, *SD* = 18.00). The principal component analysis is a statistical method that distills a small set of variables down to several variables that reflect the important features without losing any key information. Demographic characteristics were measured by the percentage of Latinos (*M* = 19.44, *SD* = 25.06), the percentage of Asians (*M* = 4.00, *SD* = 8.60), and percentage of foreign-born residents (*M* = 13.00, *SD* = 15.10).

#### Neighborhood collective efficacy

Neighborhood collective efficacy was measured by the Informal Social Control subscale (five items) and Social Cohesion (five items) subscale at children’s age 3, which were two well-developed scales that are commonly used in previous studies [49]. Caregivers rated their neighborhood collective efficacy using a 4-point scale. Questions included “This is a close-knit neighborhood” and “People in this neighborhood do not share the same values” etc. Two items were reverse-coded in each wave, and sum scores for the two factors were calculated, with higher scores indicating better social cohesion and social control. Cronbach’s alpha values indicated good internal consistency for these subscales (social cohesion: α = .88; social control: α = .80).

#### Control variables

The focal child’s demographic information at age 3 was controlled, including gender (0 = *male*, 1 = *female*), race (White, Black, Hispanic, or other), primary caregiver’s education level, poverty level, parental marital status, and number of children in the household. Categorical variables were dummy coded. Since child maltreatment, maternal drug use, and maternal mental health were suggested as critical predictors for child behavioral problems in previous studies [17,59,60], child maltreatment experiences at age 3 were controlled in this study. Sum scores of psychological aggression (α = .59), physical assault (α = .65), and neglect (α = .58) were calculated at age 3, with higher scores indicating more severe maltreatment experiences. The internal consistency of the three maltreatment types was acceptable based on Hinton et al.’s [61] research. Mother’s drug use, anxiety, and depression were measured as binary variables at child age 3.

### Analytic strategy

Parallel-process growth curve modeling was conducted using Mplus 8.0 [62]. Parallel-process growth curve modeling was a joint analysis of both the processes of internalizing and externalizing symptoms from early childhood to adolescence. This model consists of two steps. First, the shape of the two processes had to be determined, and then the covariates were added. In this study, both linear and quadratic unconditional parallel-process growth curve models were examined first. Bayesian information criterion (BIC) and Akaike information criterion (AIC) were used to choose the best-fitting model [63]. Smaller BIC and AIC values indicated a better-fitting model. Then, a conditional parallel-process growth curve model was estimated by adding predictors (neighborhood structural and collective efficacy) and control variables (gender, race, caregiver’s education level, poverty level, parental marital status, number of children in household, psychological aggression, physical assault, neglect, mother’s drug use, anxiety, and depression). The following model fit indexes were tested: comparative fit index (CFI), root mean square error of approximation (RMSEA), and standardized root mean square residual (SRMR). CFI greater than.95, RMSEA less than or equal to.05, and SRMR less than.05 indicate a good model fit [64,65]. Missing data were handled using full information maximum likelihood estimation.

## Results

### Descriptive statistics

Slightly less than half of the focal children were female (47.80%). Approximately 48.60% of the participants were Black, 25.40% were Hispanic, and 22.00% were White. Regarding education, 34.70% of primary caregivers completed less than high school, and around 42.00% of families had household income lower than the poverty line. A third (32.10%) of mothers were married to the focal child’s biological father, and the average number of children in the household was 2.30. The average scores for psychological aggression, physical assault, and neglect at age 3 were 7.95, 6.14, and 0.37, respectively. Approximately 0.8% of the mothers used drugs, 4.60% of mothers were diagnosed with anxiety, and 20.60% of mothers were diagnosed with depression at children’s age 3. Descriptive statistics of key variables are displayed in Table 1.

**Table 1:** Descriptive statistics of observed variables (N = 2,385).

	*M* (*SD*)	%	Range
Gender (female)		47.80	
Race			
White		22.00	
Black		48.60	
Hispanic		25.40	
Other		4.00	
Primary caregiver’s education			
Less than high school		34.70	
High school or above		30.30	
College		35.00	
Household poverty level (%)			
0–99		42.00	
100–199		25.00	
200–299		13.50	
300+		19.50	
Mother married to child’s biological father		32.10	
Number of children in household	2.30 (1.34)		
Child maltreatment at age 3			
Psychological aggression	7.95 (4.87)		0–30
Physical assault	6.14 (5.04)		0–27
Neglect	0.37 (1.42)		0–30
Mother’s drug use at age 3		0.80	
Maternal anxiety at age 3		4.60	
Maternal depression at age 3		20.60	
Economic disadvantage (census tract)			
Non-Hispanic Black population	39.35 (36.58)		
Families below federal poverty line	18.03 (13.88)		
Unemployed population (age 16+)	10.27 (7.35)		
Population without bachelor’s degree	82.79 (14.83)		
Households on public assistance	7.63 (6.81)		
Ethnic heterogeneity (census tract)			
Latino	19.44 (25.06)		
Asian	4.32 (8.62)		
Foreign born	13.21 (15.09)		
Residential instability (census tract)	49.03 (23.96)		
Social cohesion	17.38 (4.93)		5–25
Social control	18.07 (6.13)		5–25
Children’s behavior problems			
Age 3 internalizing symptoms scores	2.59 (2.05)		0–13
Age 5 internalizing symptoms scores	1.44 (1.64)		0–10
Age 9 internalizing symptoms scores	1.13 (1.67)		0–14
Age 15 internalizing symptoms scores	1.90 (2.26)		0–14
Age 3 externalizing symptoms scores	9.35 (5.84)		0–32
Age 5 externalizing symptoms scores	4.75 (3.93)		0–25
Age 9 externalizing symptoms scores	3.24 (3.79)		0–34
Age 15 externalizing symptoms scores	3.84 (4.29)		0–30

### Unconditional parallel-process model

**Fig 1:**
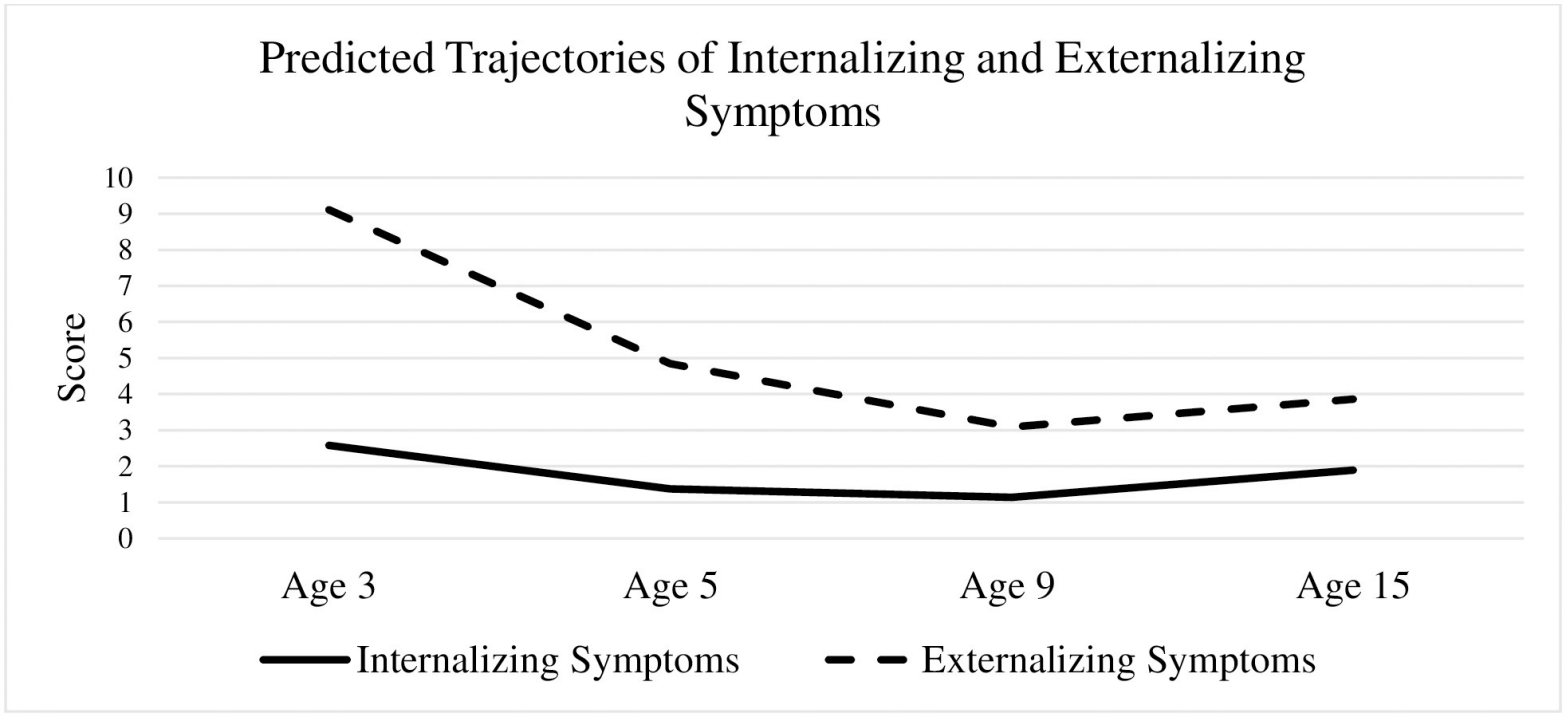
Predicted trajectories of internalizing and externalizing symptoms.

Both linear unconditional parallel-process and quadratic unconditional parallel-process models were estimated in this study. Compared with the linear unconditional parallel-process model (AIC = 115,902.30, BIC = 116,067.09), the quadratic unconditional parallel-process model had a significantly better model fit (AIC = 114,143.63, BIC = 114,390.83). The average internalizing and externalizing symptom scores at baseline were 2.58 and 9.11, respectively, and the slopes and quadratics of both internalizing and externalizing symptoms were significant (unstandardized internalizing slope = -1.72, *p* < .001; unstandardized externalizing slope = -5.53, *p* < .001; unstandardized internalizing quadratic = 0.49, *p* < .001; unstandardized externalizing quadratic = 1.26, *p* < .001; Table 2). Such findings suggested that both internalizing and externalizing symptoms decreased from age 3, reached a stationary point around age 9, and then increased thereafter to age 15 (Fig 1).

**Table 2:** Parameter estimates from unconditional parallel-process latent growth curve model.

	Estimate	Variance	1	2	3	4	5
1. Internalizing intercept	2.58***	2.38***					
2. Internalizing slope	-1.72***	1.61***	-.77***				
3. Internalizing quadratic	0.49***	0.09**	.64***	-.90***			
4. Externalizing intercept	9.11***	19.83***	.69***	-.40***	.34**		
5. Externalizing slope	-5.53***	6.26**	-.78***	.57***	.37*	-.83***	
6. Externalizing quadratic	1.26***	0.27*	.77***	-.62***	.48*	.73***	-.89***

*Note*. The growth parameter estimates are unstandardized and the correlations are standardized for ease of interpretation.

*p < .05.

**p < .01.

***p < .001.

The cross-domain correlations among all interceptions, linear slopes, and quadratics of both internalizing and externalizing symptoms were significant (as shown in Table 2). For example, the internalizing slope was positively related to the externalizing quadratic (*r* = .77, *p* < .001), suggesting that children showing a faster increase in internalizing symptoms had a higher quadratic increase in externalizing symptoms. The initial level of internalizing symptoms was positively related to the initial level of externalizing symptoms (*r* = .69, *p* < .001), indicating that children with a higher initial level of internalizing symptoms were more likely to experience a higher initial level of externalizing symptoms as well. The linear slopes and quadratic parameters of internalizing and externalizing symptoms were also positively correlated (slope: *r* = .57, *p* < .001; quadratic: *r* = .48, *p* = .02), suggesting that the decline and increase of internalizing symptoms over time were correlated with similar changes in externalizing symptoms. Notably, there were significant negative relationships between the initial level of internalizing symptoms and the slope of externalizing symptoms (*r* = -.78, *p* < .001) and between the slope of internalizing symptoms and the intercept of externalizing symptoms (*r* = -.40, *p* < .001), suggesting that children with a higher initial level of internalizing symptoms showed slower changes in externalizing symptoms over time and vice-versa for children with a higher initial level of externalizing symptoms.

### Conditional parallel-process model

The influences of two neighborhood factors on the trajectories of internalizing and externalizing symptoms are shown in Table 3. The conditional parallel-process model showed a good model fit: CFI = .99, RMSEA = .03 (90% CI = 0.02, 0.03), SRMR = .01. The results show that children living in neighborhoods with economic disadvantage had higher initial levels of both internalizing and externalizing symptoms (internalizing symptoms: *b* = 0.14, 95% CI = 0.03, 0.25, *p* = .02; externalizing symptoms: *b* = 0.42, 95% CI = 0.11, 0.72, *p* = .009). Higher level of economic disadvantage predicted changes in internalizing symptoms (*b* = -0.15, 95% CI = -0.30, 0.00, *p* = .04). This result suggests that in neighborhoods with higher levels of economic disadvantage, the initial level of children’s internalizing symptoms was higher, and the trend of internalizing symptoms had a steeper decrease. Children living in neighborhoods with a higher level of social cohesion had lower initial levels of both internalizing and externalizing symptoms (internalizing symptoms: *b* = -0.03, 95% CI = -0.05, -0.10, *p* = .004; externalizing symptoms: *b* = -0.14, 95% CI = -0.19, -0.09, *p* < .001). A higher level of social cohesion was positively associated with changes in externalizing symptoms (*b* = 0.06, 95% CI = 0.01, 0.12, *p* = .03). This result indicates that in communities with a higher level of social cohesion, children’s initial level of externalizing symptoms was lower, and the trend of externalizing symptoms had a steady decrease.

**Table 3:** Predictors of latent growth factors of internalizing and externalizing symptoms.

	Internalizing Symptoms						Externalizing Symptoms					
	Intercept		Slope		Quadratic		Intercept		Slope		Quadratic	
	*b*	95% CI	*b*	95% CI	*b*	95% CI	*b*	95% CI	*b*	95% CI	*b*	95% CI
Neighborhood Structural factors												
Economic disadvantage	0.14*	0.03, 0.25	-0.15*	-0.30, 0.00	0.03	-0.02, 0.08	0.42**	0.11, 0.72	-0.14	-0.47, 0.20	0.03	-0.07, 0.12
Residential instability	-0.02	-0.47, 0.43	0.15	-0.44, 0.73	-0.03	-0.22, 0.16	-0.41	-1.64, 0.82	-0.57	-1.90, 0.76	0.26	-0.13, 0.65
Demographic characteristics	0.08	-0.04, 0.19	0.04	-0.11, 0.19	-0.03	-0.07, 0.02	-0.10	-0.42, 0.21	0.23	-0.10, 0.57	-0.09	-0.19, 0.01
Neighborhood collective efficacy												
Social cohesion	-0.03**	-0.05, -0.10	0.01	-0.02, 0.03	-0.00	-0.01, 0.01	-0.14***	-0.19, -0.09	0.06*	0.01, 0.12	-0.01	-0.03, 0.01
Social control	0.00	-0.01, 0.02	-0.00	-0.02, 0.02	0.00	-0.01, 0.01	-0.01	-0.05, 0.04	-0.01	-0.06, 0.03	0.01	-0.01, 0.02
***Control variables***												
Gender	-0.01	-0.17, 0.15	-0.30**	-0.52, -0.09	0.13***	0.06, 0.20	-0.61**	-1.06, -0.17	-0.20	-0.69, 0.28	0.08	-0.06, 0.23
Race												
Black	0.27*	0.03, 0.51	-0.66***	-0.97, -0.35	0.09	-0.01, 0.19	-0.83*	-1.47, -0.18	-0.09	-0.80, 0.61	0.05	-0.15, 0.26
Hispanic	0.22	-0.06, 0.50	-0.22	-0.59, 0.14	-0.02	-0.14, 0.10	0.18	-0.58, 0.94	-0.78	-1.61, 0.04	0.15	-0.09, 0.39
Mother’s education level (above high school)	-0.07	-0.25, 0.11	0.12	-0.11, 0.36	-0.04	-0.12, 0.04	-0.23	-0.72, 0.27	0.40	-0.14, 0.94	-0.12	-0.27, 0.04
Mother married to child’s biological father	-0.01	-0.35, 0.33	0.21	-0.24, 0.65	-0.10	-0.24, 0.05	-0.60	-1.52, 0.32	0.29	-0.71, 1.29	-0.05	-0.35, 0.24
Number of children in household	-0.03	-0.10, 0.03	-0.05	-0.13, 0.04	0.02	-0.01, 0.05	0.00	-0.18, 0.18	-0.06	-0.25, 0.13	0.04	-0.02, 0.09
Household poverty level	-0.21***	-0.28, -0.14	0.09*	0.00, 0.18	-0.02	-0.05, 0.01	-0.35***	-0.53, -0.16	0.12	-0.09, 0.32	-0.03	-0.09, 0.03
Emotional maltreatment	0.39*	0.07, 0.71	-0.24	-0.66,0.18	0.06	-0.08, 0.19	2.37***	1.51, 3.23	-2.03***	-2.98, -1.07	0.44**	0.16, 0.72
Physical maltreatment	0.33**	0.09, 0.56	-0.21	-0.52, 0.11	0.05	-0.05, 0.15	2.17***	1.53, 2.82	-1.35***	-2.06, -0.65	0.29**	0.08, 0.49
Neglect	0.81***	0.54, 1.08	-0.36*	-0.71, -0.01	0.07	-0.04, 0.18	1.90***	1.16, 2.64	-0.80*	-1.59, 0.00	0.09	-0.14, 0.32
Mother’s drug use	-0.89	-1.90, 0.12	1.43*	0.10, 2.77	-0.34	-0.76, 0.09	-0.38	-3.13, 2.37	0.05	-2.96, 3.05	0.004	-0.87, 0.88
Maternal anxiety	0.28	-0.12, 0.69	0.05	-0.48, 0.58	-0.02	-0.19, 0.15	0.21	-0.90, 1.32	-0.41	-1.62, 0.81	0.10	-0.26, 0.45
Maternal depression	0.44***	0.22, 0.65	-0.22	-0.50, 0.07	0.09*	0.00, 0.19	1.54***	0.94, 2.13	-0.59	-1.23, 0.05	0.13	-0.05, 0.32

## Discussion

This study captured the co-development and cross-domain relations of internalizing and externalizing symptoms from early childhood to adolescence and the long-term influences of different neighborhood factors on such trajectories, which enriches the understanding of child behavior problems from a macro-level perspective. Co-occurring quadratic trajectories were identified for internalizing and externalizing symptoms from ages 3 to 15. Both internalizing and externalizing symptoms decreased from ages 3 to 9 and slightly increased afterward, which supports Godinet and Berg’s findings [34]. Compared to the trajectory of externalizing symptoms, the trajectory of internalizing symptoms was relatively stable over time [24]. The decrease in internalizing and externalizing symptoms from ages 3 to 9 can be explained by the development of children’s cognitive abilities [66]. As time goes by, children develop better cognitive abilities, which can help them regulate impulses and express emotions more effectively. However, once children approach puberty (after age 9), due to a widening array of social and environmental stressors compared to a relatively stress-free childhood, both internalizing and externalizing symptoms increase during this developmental period [67]. Another potential explanation for this U-shape is that children may accept interventions after parents recognize their children’s behavioral problems, but the effects of interventions may not last long enough.

Bidirectional relationships were found across domains in this study, which means both the initial levels of and changes in internalizing and externalizing symptoms were correlated to each other. The initial level of internalizing symptoms was associated with the nonlinear pattern of externalizing symptoms, and the initial level of externalizing symptoms was associated with the nonlinear pattern of internalizing symptoms. This finding supports the theoretical idea that co-occurrence is a common and normative pattern of internalizing and externalizing symptoms in childhood and adolescence [25,68]. Shared genetic and risk factors, such as the failure to achieve socio-developmental milestones, were proposed to account for the presence of co-occurrence [25,38]. Because the sample of this study featured many children in single-parent families, such a reciprocal relationship between internalizing and externalizing symptoms warrants examinations in future studies in the general population.

Another important aim of this study was to examine whether two types of neighborhood factors affect the internalizing and externalizing symptoms, which provides empirical evidence for social-ecological theory. The long-term influences of neighborhood factors on individuals were examined. As expected, living in neighborhoods with severe economic disadvantage was associated with high initial levels of both internalizing and externalizing symptoms in early childhood after controlling for individual and family-level factors, which was consistently reported in previous studies [51,69,70]. Neighborhood poverty may entail a lack of resources, positive exemplars, and services that are essential for young children and their parents and further increase the development of early onset internalizing and externalizing symptoms [71].

Interestingly, in conflict with previous research [40,52], current findings suggest that neighborhood economic disadvantage only affects the long-term trend of internalizing symptoms but not externalizing symptoms. This finding suggests that children living in economically disadvantaged neighborhoods show dramatic changes in internalizing symptoms from early childhood to adolescence. Economic disadvantage had strong early onset influences on internalizing symptoms and such influences decreased over time. Thus, children living in neighborhoods with economic disadvantage in early developmental stages can experience a faster decrease in internalizing symptoms. Some research pointed out that living in economically disadvantaged neighborhoods is associated with decreases in children’s self-esteem and self-confidence [40], which may lead to a further increase in internalizing symptoms [40].

Social cohesion also plays a significant role in the trajectory of internalizing and externalizing symptoms. According to various empirical studies [53,72,73], better social cohesion is strongly associated with lower levels of internalizing and externalizing symptoms. Moreover, social cohesion had long-term influences on changes in externalizing symptoms but not internalizing symptoms, which is inconsistent with previous studies [54,74]. A potential explanation is that compared to short-term or onset influences, social cohesion is more likely to express long-term influences because of extensive exposure to the neighborhood [53]. Moreover, compared to internalizing symptoms, externalizing symptoms are more noticeable by neighbors. Therefore, social cohesion may show stronger influences on externalizing symptoms. Also, living in neighborhoods with higher social cohesion provides parents with more social support, reduces their parenting stress, and builds positive models for children, which may decrease children’s externalizing symptoms [53,75].

Finally, nonsignificant findings of other neighborhood structural factors (residential instability and demographic characteristics) and social control were presented in this study, which warrant future examination. Considering rental-occupied housing has become a trend in recent decades and the diversity of the population in the United States, the influences of residential instability and demographic characteristics may be different. As for social control, since the concept reflects residents’ willingness to stop misbehaviors, some studies mentioned a possibility that social control makes residents less likely to intervene in “private” behaviors, which happen more often in families. Thus, future research on social control is needed to specify its influences.

### Limitations

The primary limitation of this study is that the collected data solely relied on the parents’ reports of internalizing and externalizing symptoms, which may increase the bias of the measurement of internalizing and externalizing symptoms. Second, the study involved many children from single-parent families, which limited the generalizability of this study. The findings of this study cannot be generalized to the general population. Third, because this study employed a variable-centered approach, it did not capture significant individual differences regarding their developmental trajectories. Future research utilizing a person-centered approach could provide additional valuable insights into this topic.

### Conclusions and implications

Despite these limitations, the results of this study can significantly benefit interventions and preventive efforts regarding child behavioral problems. Consistent with the developmental psychopathology perspective, this study confirmed the co-occurrence of internalizing and externalizing symptoms and how they affect each other across domains. Practitioners should frequently check the comorbidity of such symptoms in different developmental stages. Providing timely treatments for internalizing and externalizing symptoms among young children may positively change their developmental trajectories. In particular, considering the highest level of externalizing symptoms was found at age 3, treatments for early childhood externalizing symptoms should be emphasized once pediatricians detect such problems among their clients. Furthermore, compared to externalizing symptoms, practitioners should look out for the slower progress of the trend of internalizing symptoms and patiently design treatment plans. In particular, practitioners should closely monitor children who have behavioral problems between ages 9 and 15, so that the relapse of internalizing and externalizing symptoms during adolescence is detected and treated.

In addition, given evidence of the long-term influences of economic disadvantage on internalizing symptoms, future interventions and policy regarding neighborhood poverty are emphasized by this study. The population living in high-poverty neighborhoods has rapidly increased since 2000 [76]. Since children’s exposure to neighborhoods featuring high economic disadvantage is pervasive, living in such neighborhoods brings substantial risk regarding internalizing symptoms. Therefore, policies and interventions that reduce neighborhood poverty are needed urgently. For example, the Moving to Opportunity program that provides affordable housing in affluent neighborhoods to families living in disadvantaged neighborhoods significantly and persistently improved such families’ living environment. More similar programs will further benefit the development of the next generation. Similarly, to improve social cohesion, community-based intervention and prevention efforts that improve neighborhood connections and support among families and peers should be promoted to benefit children’s healthy development. Moreover, urban design and land use should also be considerate about improving the positive influences of social cohesion on future residents. More community activity spaces that help promote connections among residents can benefit child health and well-being [77,78].
